# Bisphenol A Exposure Is Associated with *in Vivo* Estrogenic Gene Expression in Adults

**DOI:** 10.1289/ehp.1103809

**Published:** 2011-08-10

**Authors:** David Melzer, Lorna Harries, Riccardo Cipelli, William Henley, Cathryn Money, Paul McCormack, Anita Young, Jack Guralnik, Luigi Ferrucci, Stefania Bandinelli, Anna Maria Corsi, Tamara Galloway

**Affiliations:** 1 Epidemiology and Public Health, and; 2 Institute of Biomedical and Clinical Sciences, Peninsula College of Medicine and Dentistry, University of Exeter, Exeter, United Kingdom; 3 Biosciences, College of Life and Environmental Sciences, University of Exeter, Exeter, United Kingdom; 4 Brixham Environmental Laboratory, AstraZeneca UK Ltd., Brixham, United Kingdom; 5 Laboratory of Epidemiology, Demography, and Biometry, an; 6 Clinical Research Branch, National Institute on Aging, Baltimore, Maryland, USA; 7 InCHIANTI Group, Piero Palagi Hospital, Florence, Italy

**Keywords:** bisphenol A, endocrine disruption, estrogen receptor-β, estrogen-related receptor-α, human biomonitoring, InCHIANTI, toxicogenomics

## Abstract

Background: Bisphenol A (BPA) is a synthetic estrogen commonly used in polycarbonate plastic and resin-lined food and beverage containers. Exposure of animal and cell models to doses of BPA below the recommended tolerable daily intake (TDI) of 50 μg/kg/day have been shown to alter specific estrogen-responsive gene expression, but this has not previously been shown in humans.

Objective: We investigated associations between BPA exposure and *in vivo* estrogenic gene expression in humans.

Methods: We studied 96 adult men from the InCHIANTI population study and examined *in vivo* expression of six estrogen receptor, estrogen-related receptor, and androgen receptor genes in peripheral blood leukocytes.

Results: The geometric mean urinary BPA concentration was 3.65 ng/mL [95% confidence interval (CI): 3.13, 4.28], giving an estimated mean excretion of 5.84 μg/day (95% CI: 5.00, 6.85), significantly below the current TDI. In age-adjusted models, there were positive associations between higher BPA concentrations and higher *ESR2* [estrogen receptor 2 (ER beta)] expression (unstandardized linear regression coefficient = 0.1804; 95% CI: 0.0388, 0.3221; *p* = 0.013) and *ESRRA* (estrogen related receptor alpha) expression (coefficient = 0.1718; 95% CI: 0.0213, 0.3223; *p* = 0.026): These associations were little changed after adjusting for potential confounders, including obesity, serum lipid concentrations, and white cell subtype percentages. Upper-tertile BPA excretors (urinary BPA > 4.6 ng/mL) had 65% higher mean *ESR2* expression than did lower-tertile BPA excretors (0–2.4 ng/mL).

Conclusions: Because activation of nuclear-receptor–mediated pathways by BPA is consistently found in laboratory studies, such activation in humans provides evidence that BPA is likely to function as a xenoestrogen in this sample of adults.

Bisphenol A (BPA) is a synthetic compound that is suspected to act as an endocrine disruptor (i.e., a compound capable of causing dysfunction to hormonally regulated body systems) (Talsness et al. 2009). It was originally synthesized as a synthetic estrogen (Dodds and Lawson 1936). It is used extensively as a monomer in polycarbonate plastics and in the epoxy resins that are used to line food and beverage containers and is one of the world’s highest-production-volume chemicals (Ritter 2011). Ubiquitous exposure to BPA is believed to occur mainly through the diet, with additional contributions from dental sealants, dermal exposure, and inhalation of household dusts. BPA metabolites have been reported in the urine of > 90% of people in representative population samples in the United States and Europe (Calafat et al. 2008; Galloway et al. 2010).

Whether BPA can cause human health effects is a matter of some debate. There has been concern about the potential for a relationship between BPA and negative health effects, including increases in abnormal penile/urethra development in males, early sexual maturation in females, an increase in neurobehavioral problems such as attention deficit–hyperactivity disorder (ADHD) and autism, an increase in childhood and adult obesity and type 2 diabetes, and an increase in hormonally mediated cancers, such as prostate and breast cancers (reviewed by Hengstler et al. 2011; vom Saal et al. 2007). Cross-sectional epidemiological studies have shown higher BPA exposure to be associated with adverse health effects in the general adult population. In a study of 1,455 respondents in the 2003–2004 U.S. population-representative National Health and Nutrition Examination Survey (NHANES), higher urinary BPA concentrations were associated with cardiovascular disease diagnoses and with diagnosed diabetes but not with other common diseases, suggesting specificity of the reported findings (Lang et al. 2008). In a further study of data from NHANES 2005/2006, higher BPA concentrations were again associated with coronary heart disease, providing independent replication of the findings (Melzer et al. 2010). Higher exposure to BPA has also been associated with reproductive and developmental abnormalities. In a study of 249 mothers and their children, prenatal urinary BPA concentrations in mothers were prospectively associated with externalizing behavior scores among their children when measured at 2 years of age (Braun et al. 2009). A positive association was also shown between BPA exposure and recurrent miscarriage in a prospective study of 67 women (Sugiura-Ogasawara et al. 2005). The mechanisms underlying these potential health effects remain to be determined.

Most studies of the health effects of BPA have focused on its estrogenic activity because it is widely documented to function as an agonist of certain estrogen receptors (ERs) (Lee et al. 2003) and as an androgen antagonist and to suppress aromatase activity (Bonefeld-Jørgensen et al. 2007). Additional receptor-mediated biological activities, including binding to the orphan estrogen-related receptor ERRγ (Okada et al. 2008), thyroid hormone disruption (Moriyama et al. 2002), altered pancreatic β-cell function (Ropero et al. 2008), and obesity-promoting effects (Newbold et al. 2008), have been reported in different model systems. Many of these effects are already detectable in the nanomolar range, prompting calls for a revision to the current tolerable daily intake (TDI) of 50 μg/kg/day. However, until now, there has been no evidence that BPA at these low levels exerts significant biological effects in humans, and hence the TDI has remained unaltered (European Food Safety Authority 2010).

A recent cross-sectional examination of circulating sex hormone concentrations in 307 men showed higher BPA levels to be associated with changes in total testosterone concentrations [β = 0.046; 95% confidence interval (CI): 0.015, 0.076; *p* = 0.004 in fully adjusted models] (Galloway et al. 2010). There was no significant trend in 17β-estradiol levels with higher BPA in men, although an earlier study of 167 men recruited through an infertility clinic used multiple adjusted regression models to show BPA concentrations in urine to be inversely associated with the estradiol:testosterone ratio (Meeker et al. 2010). Plausible explanations for these endocrine changes include altered expression of hormone-responsive genes. To date there is no *in vivo* evidence for changes in sex-hormone–responsive gene expression associated with human exposure to BPA.

Here, we aimed to test the hypothesis that exposure to BPA would be associated with changes in the *in vivo* expression of estrogen- and androgen-responsive genes. To do this, we conducted a cross-sectional study to characterize six candidate estrogen- or androgen-related transcripts for differential *in vivo* expression in response to BPA exposure. The study population was selected from the InCHIANTI study, a large European population representative sample based in Chianti, Italy.

## Materials and Methods

*Study population.* The InCHIANTI study, a prospective population-based study of Italian adults (InCHIANTI 2011), was designed to identify risk factors for mid- and late-life morbidity in urban and rural populations and has been described extensively elsewhere (Ferrucci et al. 2000). InCHIANTI is performed in two sites: Greve in Chianti (11,709 inhabitants) and Bagno a Ripoli (Village of Antella, 4,704 inhabitants). The final study population included 1,453 persons (age range 20–102 years) stratified across age ranges using a multistage sampling process, with a response rate of 91.6% from the baseline interview. Subjects and specimens selected for the present study were those with the most adequate RNA and urine specimens in the 2008/2009 follow-up, and ≤ 76 years of age, in line with previous work. Women were excluded from this analysis because of cyclic hormonal variations in premenopausal subjects. The Instituto Nazionale Riposo e Cura Anziani Institutional Review Board (Florence, Italy) provided ethical approval. All participants gave informed (or surrogate) consent.

*Sample collection.* Participants who consented to give a blood sample were also asked to provide a spot morning urine sample, which was stored at –20°C until further analysis. First thing in the morning on the day of the study visit, after participants had been sedentary for 15 min, fasting blood samples were collected for routine blood examination, and peripheral blood specimens preserving *in vivo* RNA expression were collected using PAXgene technology (Debey-Pascher et al. 2009).

*Analysis of urinary BPA concentrations.* Samples were analyzed at the Brixham Environmental Laboratory Division of Analytical Chemistry (a division of AstraZeneca PLC; Brixham, UK) in compliance with Good Laboratory Practice, EU Directive 88/32/EEC (United Kingdom 2004). BPA ingested in humans is almost completely metabolized and rapidly excreted, so urine is considered the most appropriate matrix for assessment of exposure (Calafat et al. 2005). As part of our extensive Good Laboratory Practice–compliant quality control, we included reagent blanks and confirmed that samples stored for up to 10 years contained predominantly metabolized compound, confirming minimal leaching of BPA from collection or storage vessels during this time. BPA concentrations were measured in spot urine samples by liquid chromatography–mass spectrometry. Total (free and conjugated) urinary concentrations of BPA were obtained using online, solid-phase extraction coupled with high-performance liquid chromatography–isotope dilution tandem mass spectrometry with peak focusing, as described previously (Galloway et al. 2010). Calibration was linear from 0.50 to 100 μg/L (*R*^2^ > 0.996), limit of detection was < 0.50 ng/mL BPA, the limit of quantification was 0.50 ng/mL BPA, and the lowest calibration standard gave a signal height:noise ratio > 10 (relative standard deviations < 20%, all other standards < 15%).

*Gene expression by real-time reverse-transcriptase polymerase chain reaction (RT-PCR).* Blood leukocytes were used for transcript analysis because they are convenient and available and because they provide sufficient power in large cohorts where access to other tissues is lacking. Because BPA is metabolized in the intestines and liver to form predominantly BPA-monoglucuronide, which is passed through the bloodstream to the kidney, exposure of leukocytes to BPA and/or its metabolites is inevitable. To test the hypothesis that exposure to BPA would be associated with changes in the expression of estrogen- and androgen-responsive genes, we correlated BPA levels as a continuous trait with the expression of ER, androgen receptor (AR), and estrogen-related receptor (ERR) genes by quantitative real-time PCR in a subset of 100 male subjects. These genes were chosen because the nuclear hormone receptors they encode are transcription factors that control essential developmental and physiological pathways and because activation of these nuclear-receptor–mediated pathways by BPA is consistently found in laboratory studies.

Total RNA (100 ng) was reversed transcribed in 20 μL reactions using the Superscript III VILO kit (Invitrogen, Paisley, UK), according to the manufacturer’s instructions.

The expression levels of *ESR1* (estrogen receptor 1; ERα), *ESR2* [estrogen receptor 2 (ER beta); ERβ], *ESRRA* (estrogen related receptor alpha; ERRα), *ESRRB* (estrogen related receptor beta; ERRβ), *ESRRG* (estrogen related receptor gamma), and *AR* (androgen receptor) genes were then assessed relative to the endogenous control genes *GUSB* (glucuronidase, beta) and *ACTB* (actin, beta; β-actin) on the TaqMan Low Density Array (TLDA) platform (Applied Biosystems, Foster City, CA, USA). Probes were inventoried with Applied Biosystems assays Hs01046812_m1, Hs01100358_m1, Hs01584024_m1, Hs00155006_m1, Hs00907244_m1, Hs99999908_m1, and Hs03023943_g1 for *ESR1*, *ESR2*, *ESRRB*, *ESRRG*, *AR*, *GUSB*, and *ACTB* genes, respectively. These probes were chosen because they are documented to pick up all isoforms and splice variants for the genes of interest.

The expression of the *ESRRA* gene was assessed by the use of a custom assay (probe and primer sequences available on request). Reaction mixes included 50 μL 2× TaqMan universal master mix (no AMPerase; Applied Biosystems), 40 μL distilled H_2_O, and 10 μL cDNA template per TLDA loading port. PCR amplifications were performed on the ABI 7900HT platform (Applied Biosystems). Cycling conditions were 50°C for 2 min, 94.5°C for 10 min followed by 40 cycles of 97°C for 30 sec and 57.9°C for 1 min. The expression of each gene was measured in triplicate for each sample. Gene expression relative changes were quantified using the 2^–ΔΔCt^ method (Livak and Schmittgen 2001) relative to the geometric mean of the endogenous controls listed above using the StatMiner relative quantification software for high-throughput integrated analysis of TLDA data (Integromics, Grenada, Spain).

*Statistical analysis.* We assessed the association of candidate gene expression levels with urinary BPA concentration by multivariable linear regression. Data were adjusted for potential confounding factors that could influence BPA exposure or candidate gene expression: age (reported in years at the last birthday and used as a continuous variable); body mass index (BMI) calculated as weight in kilograms divided by height in meters squared; waist circumference (as a continuous trait); highest level of education attained (in four categories: none/elementary, secondary, high school, and university/professional); low-density lipoprotein (LDL) cholesterol (milligrams per deciliter); triglycerides (milligrams per deciliter); and study site [individuals were drawn from a rural village (Greve) and an urban population (Bagno a Ripoli)]. Models were also adjusted for the percentage of neutrophils (neutrophil%), lymphocytes (lymphocyte%), monocytes (monocyte%), and eosinophils (eosinophil%) [the percentage of basophils (basophil%) was not added because the cell subtype percentages would have equaled 100%].

The expression value of each of the target genes was not normally distributed, and we used natural log transformation when gene expression was considered as a dependent variable. In all analyses, an upper age cutoff was 76 years to minimize the problem of comorbidity. Data analysis was performed using STATA (version 10 SE; StataCorp LP, College Station, TX, USA); *p* < 0.05 was considered significant.

We used generalized additive models with penalized cubic regression splines (Wood 2006) to explore the functional form of the relationship between candidate gene expression levels and urinary BPA concentration. Linearity of the relationship between log-transformed expression level and log-transformed BPA concentration was assessed by visual inspection of the estimated spline functions and by examining the estimated degrees of freedom (edf) for the smoothed BPA term. Values of the edf close to 1 were taken as evidence of linearity. Adjustment was made for the same potential confounding factors that were included in the multivariable linear regression models. The prediction error criterion for smoothness selection was generalized cross-validation. Robustness of the smoothness selection was assessed by making comparisons with the use of maximum likelihood estimation. The spline models were fitted using R statistical software using the mgcv package for generalized additive modeling (version 2.12.1; R Project for Statistical Computing 2010).

## Results

The sample (*n* = 96; Table 1) had a mean age of 58.3 years (range, 32–76 years) and a mean (± SD) BMI of 27.8 ± 4.1 kg/m^2^. The geometric mean urinary BPA concentration was 3.65 ng/mL (95% CI: 3.13, 4.28) ranging from 0.73 to 56.94 ng/mL (limit of detection < 0.5 ng/mL). The distribution was skewed, with a 10th percentile of 1.3 ng/mL and a 90th percentile of 10.4 ng/mL. The estimated mean excretion was 5.84 μg/day (95% CI: 5.00, 6.85).

**Table 1:** Characteristics of the sample (*n* = 96).

Characteristic	Mean ± SD (range)*a*
Age (years)	58.3 ± 15.2 (32–76)
Site (%)	
Greve	38.4
Bagno a Ripoli	61.5
Education (%)	
None/elementary	22.9
Secondary school	26.0
High school	35.4
Professional/university	16.6
BMI (kg/m^2^)	27.8 ± 4.1 (18.38–42.99)
LDL cholesterol (mg/dL)	125.4 ± 29.8 (60–220)
Triglycerides (mg/dL)	137.3 ± 75.3 (45–469)
Neutrophils%	55.2 ± 9.7 (26.2–79.1)
Lymphocytes%	32.8 ± 9.2 (9.1–59.9)
Monocytes%	8.4 ± 2 (4.3–21.3)
Eosinophils%	3 ± 1.7 (0.1–10.3)
Basophils%	0.5 ± 0.2 (0.1–1.4)
**a**Values shown are mean ± SD (range) except where indicated.	

The expression of transcripts associated with sex-hormone–related signaling was quantified by real-time RT-PCR (Table 2). Expression of *ESRRG* was not detected in our samples. There was only one significant correlation of expression intensities between probes: between *ESR1* and *ESR2* (pairwise correlation = 0.24; *p* = 0.02). We obtained valid expression intensity measures for 96 men for the *ESR2* gene and 83 men for the *ESRRA* gene (Table 2). BPA concentrations in the 96 respondents with successful *ESR1* expression measures were no different from the remaining 55 respondents < 76 years of age (age-adjusted regression with log-transformed BPA concentration: unstandardized linear regression coefficient = 0.012; 95% CI: –0.114, 0.138; *p* = 0.848) for which measured BPA values were available.

**Table 2:** Expression characteristics of the tested estrogen and androgen target genes.

Target gene	Assay ID*a*	Accession number*b*	*n*	Mean ± SD (range)
*ESR1*		Hs01046812_m1		NM_000125		96		1.21 ± 0.535 (0.365–3.165)
*ESR2*		Hs01100358_m1		NM_001040275		96		1.294 ± 0.899 (0.167–5.585)
*ESRRA*		Hs01067166_g1		NM_004451		83		0.882 ± 0.33 (0.105–1.991)
*ESRRB*		Hs01584024_m1		NM_004452		96		2.974 ± 2.434 (0.000–10.363)
*ESRRG*		Hs00155006_m1		NM_206595				Not expressed
*AR*		Hs00907244_m1		NM_000044.2		96		1.232 ± 0.673 (0.188–3.295)
**a**TaqMan Gene Expression assay identification number. **b**Accession numbers from the National Center for Biotechnology Information (2011).								

Using urinary BPA concentrations as a continuous variable, we tested linear associations between BPA and gene expression. In age-adjusted regression models of log-transformed BPA concentrations against log-transformed expression levels (Table 3), we observed positive associations with *ESR2* (ERβ; coefficient = 0.1804; 95% CI: 0.0388, 0.3221; *p* = 0.013) and *ESRRA* (ERRα, coefficient = 0.1718, 95% CI: 0.0213, 0.3223, *p* = 0.026) but not with *ESR1*(ERα), *ESRRB*(ERRβ), or *AR*.

**Table 3:** Estimates for the associations between natural log of urinary BPA concentrations and gene expression intensity (log transformed), in age-adjusted and fully adjusted*a* regression models.

Age-adjusted model					Fully adjusted model				
Gene	Coefficient (95% CI)	*p*-Value	Std β	Coefficient (95% CI)	*p*-Value	Std β
*ESR1*		–0.0657 (–0.1815, 0.0500)		0.262		–0.117		–0.1071 (–0.2205, 0.0063)		0.064		–0.1909
*ESR2*		0.1804 (0.0388, 0.3221)		0.013		0.231		0.1387 (0.001, 0.2764)		0.048		0.1775
*ESRRA*		0.1718 (0.0213, 0.3223)		0.026		0.250		0.1886 (0.0324, 0.3448)		0.019		0.2699
*ESRRB*		–0.2816 (–1.3969, 0.8337)		0.617		–0.054		–0.4857 (–1.6669, 0.6955)		0.416		–0.0925
*ESRRG*		ND						ND				
*AR*		0.0115 (–0.1404, 0.1634)		0.881		0.016		0.0925 (–0.0646, 0.2495)		0.245		0.1285
Abbreviations: ND, not detected; Std, standardized. **a**Full adjustment included age, BMI, study site, educational attainment, and LDL cholesterol and triglyceride concentrations, plus percentages of neutrophils, lymphocytes, monocytes, and eosinophils.												

In models additionally adjusted for previously suggested confounders (Sharpe 2010) (BMI, LDL cholesterol and triglyceride concentrations, study site, and educational attainment—a proxy for social position) and white cell subtype percentages, the results were little changed: for *ESR2*, coefficient = 0.1387; 95% CI: 0.001, 0.2764; *p* = 0.048; for *ESRRA* coefficient = 0.1886; 95% CI: 0.0324, 0.3448; *p* = 0.019) (Table 4).

**Table 4:** Multiple regression model estimates for the associations between explanatory variables and natural logs of *ESR2*and *ESRRA *gene expression.

*ESR2*					*ESRRA*				
Variable	Coefficient (95% CI)	*p*-Value	Std β	Coefficient (95% CI)	*p*-Value	Std β
BPA concentration (log transformed)		0.1387 (0.001, 0.2764)		0.048		0.1775		0.1886 (0.0324, 0.3448)		0.019		0.2699
Age		–0.0169 (–0.0261, –0.0078)		< 0.001		–0.4256		0.0018 (–0.0086, 0.0122)		0.733		0.0470
BMI		0.0206 (–0.0057, 0.0468)		0.122		0.1399		–0.0023 (–0.0312, 0.0265)		0.873		–0.0194
Study site		0.2016 (–0.0098, 0.413)		0.061		0.1629		–0.218 (–0.4489, 0.0129)		0.064		–0.2087
Educational attainment												
None/elementary		1 (Reference)						1 (Reference)				
Secondary		0.1146 (–0.2084, 0.4375)		0.482		0.0834		–0.4742 (–0.8521, –0.0962)		0.015		–0.4176
High school		–0.0347 (–0.3704, 0.3011)		0.838		–0.0275		–0.099 (–0.4865, 0.2885)		0.612		–0.0929
Professional/university		0.0899 (–0.2604, 0.4403)		0.611		0.0542		–0.0379 (–0.4521, 0.3764)		0.856		–0.0268
LDL cholesterol (mg/dL)		–0.0024 (–0.006, 0.0012)		0.195		–0.117		0.0016 (–0.0022, 0.0055)		0.407		0.094
Triglycerides (mg/dL)		0.0012 (–0.0002, 0.0025)		0.095		0.1444		–0.0009 (–0.0023, 0.0006)		0.255		–0.1339
Neutrophil%		–0.3194 (–0.8618, 0.2229)		0.245		–5.1245		0.3622 (–0.2803, 1.0047)		0.265		6.5463
Lymphocyte%		–0.302 (–0.845, 0.2411)		0.272		–4.606		0.3611 (–0.2828, 1.0049)		0.267		6.1452
Monocyte%		–0.2982 (–0.8349, 0.2385)		0.272		–1.0051		0.3678 (–0.265, 1.0007)		0.250		1.5245
Eosinophil%		–0.3075 (–0.8726, 0.2575)		0.282		–0.8726		0.3624 (–0.2947, 1.0196)		0.275		1.2623
Constant		31.078 (–23.088, 85.244)		0.257				–36.1216		0.264		0
Std, standardized. “Constant” refers to the intercept term in the multiple regression model; it gives the expected log-transformed gene expression level when all other variables in the model are set to zero.												

**Figure 1:**
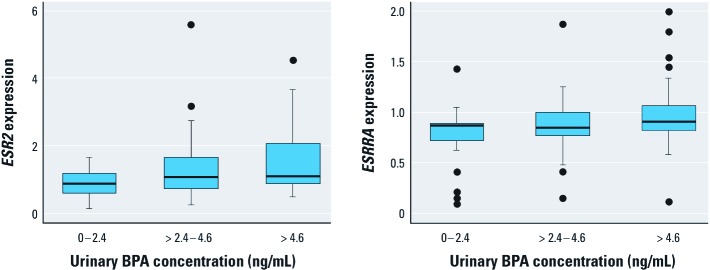
Box plot of *ESR2* and *ESRRA* probe intensity by urinary BPA concentration. Boxes extend from the 25th to the 75th percentile, horizontal bars represent the median, whiskers indicate the 10th and 90th percentiles, and outliers are represented as circles.

When using an alternative exposure metric of dividing BPA concentrations into tertiles in the fully adjusted models (Figure 1), participants in the lowest BPA exposure tertile had a geometric mean expression of *ESR2* of 0.80 IU (95% CI: 0.65, 0.99), rising to 1.32 IU (95% CI: 1.08, 1.60) in the highest tertile, a 65% increase in mean expression. For *ESRRA*, the same measures were 0.66 IU (95% CI: 0.49, 0.89) and 0.91 IU (95% CI: 0.78, 1.06), a 38% increase in mean expression of the gene.

**Figure 2:**
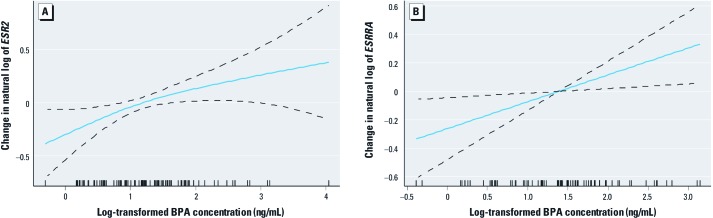
Cubic regression spline models illustrating the functional form of the relationship between log-transformed urinary BPA concentration and *ESR2* (*A*) and *ESSRA* (*B*) gene expression.

Figure 2A shows a spline plot for the change in natural log of *ESR2* expression as a function of log-transformed urinary BPA concentration. This suggests that the positive association between *ESR2* and BPA concentration is curvilinear (edf = 1.45; *p*-value for smoothed term = 0.027), with evidence of a diminishing effect as BPA concentration increases. A similar spline plot for *ESRRA* expression is shown in Figure 2B. This suggests that the relationship with BPA concentration is linear for this ERR (edf = 1.00; *p-*value for smoothed term = 0.017).

## Discussion

In this study, we aimed to assess whether increased urinary BPA concentrations were associated with changes in gene expression *in vivo* in the general adult population. We made use of a large-scale and high-quality population-representative data set for which specimens preserving *in vivo* RNA expression were available. We were able to measure *in vivo* expression of five ER, ERR, and AR genes in peripheral blood leukocytes in 96 adult men. Using urinary BPA excretion as a marker of exposure, we found that those with higher BPA exposures had higher expression of two estrogen-responsive genes, *ESR2* (ERβ) and *ESRRA* (ERRα).

These findings are important because they suggest that BPA is bioactive in the human body and that associations with hormone signaling and related disorders are biologically plausible. ERβ, which showed the strongest association with BPA exposure, is one of two ER subtypes that, along with ERα, mediates the physiological actions of estrogens (Swedenborg et al. 2009). ERβ and ERα have distinct tissue distribution, ligand specificities, and functions; ERα is predominant in the regulation of female reproduction, whereas ERβ is important in maintaining the structure and function of nonclassic target tissues, including prostate, colon, and cardiovascular and central nervous systems (Imamov et al. 2005). BPA displays estrogenic agonist activities against both ERα and ERβ subtypes *in vitro*, with relatively high ERβ selectivity (Matthews et al. 2001), consistent with our findings. The modulation by BPA of ER gene expression has previously been shown in animal models, at environmentally relevant concentrations. For example, exposure of rat prostate mesenchyme cells to 1 nM BPA led to altered ER gene expression, accompanied by modest stimulation of cell growth, with a threshold of induction around 30-fold less potent than 17β-estradiol (Richter et al. 2007).

ERRα belongs to the NR3B orphan nuclear receptor subgroup, which consists of ERRα, ERRβ, and ERRγ (Hong et al. 1999). All three ERRs show close sequence identity to the ERα DNA binding domain and also feature a conserved C-terminal domain with a putative ligand binding domain and interaction surfaces for coregulators, and a less conserved N-terminal domain (Giguère 2002). Despite this close structural homology to the ERs, estradiol does not bind to ERRα, and X-ray crystallography of the putative ligand-binding domain pocket of ERRα shows it to be almost completely occupied by side chains. This supports the suggestion that ERRα shows ligand-independent transcriptional activation and is largely dependent on its functional interaction with coregulators, including peroxisome proliferator-activated receptor-γ (PPARγ) coactivator 1α (PGC-1A) and PGC-1B for optimal gene regulation (Ranhotra 2010). In adults, ERRα is constitutively expressed in tissues that preferentially use fatty acids as energy sources, including adipose tissue, heart, and skeletal muscle, where it plays a significant role in regulating energy homeostasis and adaptive oxidative capacity (Dufour et al. 2007). These functions are thought to involve close cooperation with PGC-1A and ERRγ (Villena and Kralli 2008). Crucially, BPA binds to ERRγ with high affinity (Okada et al. 2008), and ERβ has been identified as an important regulator of PPARγ (Foryst-Ludwig et al. 2008).

Given the structural homology between ERs and ERRs, particularly in the DNA-binding domain, involvement of ERRs in estrogenic signaling pathways is not unexpected (Giguère 2002). ERRα has been proposed as a regulator of aromatase activity (Yang et al. 1998), and in turn, estradiol induces up-regulation of ERRα in some tissues (Shigeta et al. 1997). ERRα stimulation of androgen-responsive element–containing promoters illustrates the potential for cross-talk with signaling driven by other steroid hormones (Teyssier et al. 2008).

The functional relevance of changes in ERβ and ERRα expression in blood leukocytes has not been determined. Because estrogens and androgens can exert differential effects in function depending on the cell type and its stage of development, the consequences of BPA exposure on a wider range of adult reproductive and somatic tissues merits further attention (Goodman et al. 2008). However, up to 50% of expression changes in leukocytes for highly heritable *cis*-acting traits are also mirrored in other tissues such as adipose tissue, making them viable surrogates for exposure of other tissues (Emilsson et al. 2008). Human adipocytes express both ERβ and ERRα (Hugo et al. 2008), and adipocyte explants respond to both BPA and 17β-estradiol exposure in the nanomolar range by accumulating lipid. Taken together, these results are strongly suggestive of specific and targeted bioactivity of BPA *in vivo*, even if the clinical relevance, if any, of these findings is not yet clear.

One limitation of this analysis is its cross-sectional nature. Virtually all individuals are exposed, and because clinical trials to administer BPA in human subjects are ethically unacceptable, collecting longitudinal data demonstrating that BPA exposure induces gene expression changes *in vivo* is not currently achievable.

It is feasible that the increases in gene expression that we measured are associated with confounding variables that have not been accounted for in our models. For example, there are time-dependent changes in ERβ expression, both on a long-term scale, such as in fetal and postnatal development, and in short-term oscillations during the circadian cycle (Swedenborg et al. 2009). Although it is not possible to completely account for circadian cycles, all samples were taken at a similar time of day, and we restricted our analysis to men rather than women to minimize the influence of cyclic variation in endogenous hormones. Confounding variables that could affect BPA exposure include higher food intakes or obesity, which could be accompanied by incidentally higher intakes of BPA (Sharpe 2010). Our secondary analyses included adjustment for BMI and LDL cholesterol and triglyceride concentrations, and these made minimal difference to the overall results, arguing against obesity as an explanation for our findings. We found no associations between serum lipids and expression intensities of our candidate genes.

Another consideration is that we quantified BPA metabolites in urine, while gene expression was measured in blood leukocytes. BPA ingested in humans is rapidly excreted, so urine is considered the most viable biomonitoring approach for BPA, as detailed by Calafat et al. (2005). Single spot samples are limited measures of longer-term exposure, but a in study of temporal variability in urinary BPA metabolites, Mahalingaiah et al. (2008) found that a single spot sample had moderate sensitivity for predicting an individual’s tertiary categorization. Nepomnaschy et al. (2009) measured stability of BPA over 2-week intervals in first voided urine samples from 60 women and found a Spearman correlation of 0.5, indicating that within-individual BPA exposures were generally stable over periods of weeks. They also showed that the stability of BPA in long-term frozen samples is good. The stability of free BPA in urine was also confirmed by Ye et al. (2011).

The mean BPA concentration in our study was 3.65 ng/mL, and assuming an average 24-hr urine volume of 1,600 mL in adult men (Galloway et al. 2010), a 100% excretion rate, and a total blood volume of 6 L, the estimated concentration of BPA in the blood was in the low nanogram per milliliter range. The *in vitro* IC_50_ (half-maximal inhibitory concentration) for human ERβ receptor binding of BPA is in the micromolar range (Matthews et al. 2001), which would imply low ER occupancy rates. Given that functional effects of BPA on nuclear receptor expression have also been reported in both animal and human cells at this concentration, *in vitro* measurement may not be indicative of the *in vivo* situation where differential binding to carrier proteins and receptors may occur. There are no *in vivo* data on the rate at which BPA is converted to BPA-monoglucuronide and excreted from the body, only estimates, and because BPA is lipophilic with a log octanol–water partition coefficient (*K*_ow_) of 2.2–3.82, distribution to lipid-rich tissues is a possibility. This suggestion is supported by population-based half-lives for BPA calculated by Stahlhut et al. (2009) to be significantly longer than previous predictions of 6 hr.

The major metabolite of BPA, BPA-monoglucuronide, has no estrogenic activity, but oxidative cleavage of BPA to form the estrogenically active metabolite 4-methyl-2,4-bis(4-hydroxyphenyl)pent-1-ene (MBP) has been observed in rat liver. Okuda et al. (2010) reported that MBP was 500-fold more potent than BPA itself in inducing dose-dependent changes in expression of *ER*α and *ER*β mRNA. The significance of this metabolite in humans is not yet known. However, a comparison of the phase 1 metabolism of BPA in rat and human liver microsomes identified the oxidation product BPA-catechol to be a minor (~ 10%) metabolite in both species. BPA-catechol is considered to be a weak estrogen (Nakagawa and Suzuki 2001), suggesting that further investigation of the phase 1 metabolism of BPA in humans and the estrogenic potency of all metabolites is merited (Ye et al. 2011).

## Conclusion

We provide the first report of associations between BPA exposure and *in vivo* estrogenic gene expression in humans. We examined *in vivo* expression of six ER, ERR, and AR genes in peripheral blood leukocytes from 96 adult men from the InCHIANTI population study. We observed positive associations between higher urinary BPA concentrations and higher expression of two estrogen-responsive genes, encoding ERβ and ERRα. The associations remained statistically significant when adjusted for potential confounders, including obesity and serum lipid concentrations. The individuals in the upper tertile of BPA exposure showed 65% higher mean expression of the *ESR2* (ERβ) gene in peripheral blood leukocytes than did those in the lower tertile. Although the clinical significance of these results is not yet known, such activation in humans provides evidence that BPA is likely to function as a xenoestrogen in this population-representative sample of adults. This prompts a need for replication and scientific follow-up, for example, in examining the relationship between gene expression changes and protein expression and effects of BPA exposure in a wider range of estrogen-regulated target tissues.
